# Arthroscopic, histological and MRI analyses of cartilage repair after a minimally invasive method of transplantation of allogeneic synovial mesenchymal stromal cells into cartilage defects in pigs

**DOI:** 10.3109/14653249.2011.638912

**Published:** 2012-02-06

**Authors:** Tomomasa Nakamura, Ichiro Sekiya, Takeshi Muneta, Daisuke Hatsushika, Masafumi Horie, Kunikazu Tsuji, Tatsuo Kawarasaki, Atsuya Watanabe, Shuji Hishikawa, Yasuhiro Fujimoto, Hozumi Tanaka, Eiji Kobayashi

**Affiliations:** 1 Section of Orthopedic Surgery, Graduate School, Tokyo Medical and Dental University, Tokyo, Japan; 2 Section of Cartilage Regeneration, Graduate School, Tokyo Medical and Dental University, Tokyo, Japan; 3 Global Center of Excellence Program, International Research Center for Molecular Science in Tooth and Bone Diseases, Tokyo Medical and Dental University, Tokyo, Japan; 4 Swine and Poultry Research Center, Shizuoka Prefectural Research Institute of Animal Industry, Shizuoka, Japan; 5 Department of Orthopedic Surgery, Teikyo University Chiba Medical Center, Chiba, Japan; 6 Center for Development of Advanced Medical Technology, Jichi Medical University, Tochigi, Japan; 7 Division of Development of Advanced Treatment, Jichi Medical University, Tochigi, Japan

**Keywords:** *cartilage repair*, mesenchymal stromal cells, *pig*, *synovium*

## Abstract

**Background aims:**

Transplantation of synovial mesenchymal stromal cells (MSCs) may induce repair of cartilage defects. We transplanted synovial MSCs into cartilage defects using a simple method and investigated its usefulness and repair process in a pig model.

**Methods:**

The chondrogenic potential of the porcine MSCs was compared *in vitro*. Cartilage defects were created in both knees of seven pigs, and divided into MSCs treated and non-treated control knees. Synovial MSCs were injected into the defect, and the knee was kept immobilized for 10 min before wound closure. To visualize the actual delivery and adhesion of the cells, fluorescence-labeled synovial MSCs from transgenic green fluorescent protein (GFP) pig were injected into the defect in a subgroup of two pigs. In these two animals, the wounds were closed before MSCs were injected and observed for 10 min under arthroscopic control. The defects were analyzed sequentially arthroscopically, histologically and by magnetic resonance imaging (MRI) for 3 months.

**Results:**

Synovial MSCs had a higher chondrogenic potential *in vitro* than the other MSCs examined. Arthroscopic observations showed adhesion of synovial MSCs and membrane formation on the cartilage defects before cartilage repair. Quantification analyses for arthroscopy, histology and MRI revealed a better outcome in the *MSC*-treated knees than in the non-treated control knees.

**Conclusions:**

Leaving a synovial *MSC* suspension in cartilage defects for 10 min made it possible for cells to adhere in the defect in a porcine cartilage defect model. The cartilage defect was first covered with membrane, then the cartilage matrix emerged after transplantation of synovial MSCs.

## Introduction

Cartilage injuries are a common clinical problem and if left untreated may cause osteoarthritis, one of the leading causes of disability (1). Stem cell therapy for cartilage repair may be one possible strategy for improvement of cartilage injury. The candidate therapeutic cells are mesenchymal stromal cells (MSCs), which can be isolated from various mesenchymal tissues (2, 3). We have reported previously the superiority of human synovial-derived MSCs for cartilage repair (4–6) and *in vitro* expansion with autologous human serum (7).

Various methods have been used to transplant MSCs into cartilage defects, such as intra-articular injection (8,9) and the use of scaffolds (10). We have demonstrated recently that leaving the knee immobilized for 10 min immediately after delivering a suspension of synovial MSCs into the defect results in approximately 60% of the cells adhering to the defect to promote cartilage repair in rabbits (11). This ‘local adherent technique’ can be performed less invasively and without scaffolds compared with other methods.

We hypothesized that this method will also be useful in animals that are more closely related to humans. The purpose of the present study was to examine the usefulness of the local adherent technique with synovial MSCs in pigs. The knee joints of pigs are similar to those of humans in terms of size (12) and cartilage-specific properties (13). In this study, synovial MSCs were transplanted into the cartilage defect of pigs using the local adherent technique, and repaired cartilage was examined sequentially arthroscopically, histologically and by delayed gadolinium-enhanced magnetic resonance imaging of cartilage (dGEMRIC) (14,15).

## Methods

### Animals

All experiments were conducted in accordance with the institutional guidelines for the care and use of experimental animals of the Tokyo Medical and Dental University (Tokyo, Japan) and Jichi Medical University (Tochigi, Japan). Nine male and six female Mexican hairless pigs (National Livestock Breeding Center, Ibaraki, Japan) were used. They were 13 months old, on average 33.5 kg in weight, and skeletally mature, with the growth plates closed. All pigs were bred under specific pathogen-free conditions and had free access during the study period to food and water in a post-operative care cage (400 mm in width, 1210 mm in length and 1090 mm in height). One wild-type pig and one transgenic green fluorescent protein (GFP) pig (16) were used as donors for synovial MSC for transplantation. Two other pigs were also used as sources for MSCs for *in vitro* proliferation and differentiation assays. These four pigs were euthanized on the day when the tissues were harvested. Twelve other wild-type pigs were used as recipients. For GFP observation, two pigs were euthanized on the day MSCs were transplanted, and for observation of 1,1'-dioctadecyl-3,3,3',3'-tetramethylindocarbocyanine perchlorate (DiI; Molecular Probes, Eugene, OR, USA) two pigs were euthanized at 7 days after transplantation. For arthroscopic, histological and MRI analyses, three pigs were euthanized at 1 month, and five pigs were euthanized at 3 months, after transplantation.

### Cell isolation and culture

Synovial tissue was harvested from the suprapatellar pouch, which overlays the non-cartilaginous areas of the femur, through an arthrotomy of the knee. The tissue was digested in 3 mg/mL collagenase D solution (Roche Diagnostics, Mannheim, Germany) in α-minimal essential medium (αMEM; Invitrogen, Carlsbad, CA, USA) at 37°C for 3 h, filtered through a 70-μm nylon filter (Becton-Dickinson and Co., Franklin Lakes, NJ, USA) and the nucleated cells plated in a 150-cm^2^ culture dish (Nalge Nunc International, Rochester, NY, USA) in complete culture medium [αMEM containing 10% fetal bovine serum (FBS), 100 U/mL penicillin, 100 μg/mL streptomycin and 250 ng/mL amphotericin B (all from Invitrogen)] and incubated at 37°C with 5% humidified CO_2_. The medium was changed to remove non-adherent cells every 4–5 days and then cultured for 14 days as passage 0 without refeeding. To cryopreserve the cells, they were resuspended at a concentration of 2 × 10^6^ cells/mL in αMEM with 5% dimethylsulfoxide (Wako, Osaka, Japan) and 10% FBS. Aliquots of 2 mL were frozen slowly in a Cryo 1°C freezing container (Nalge Nunc International) and cryopreserved at −80°C. To expand the cells, a frozen vial of the cells was thawed, plated in 60-cm^2^ culture dishes, and incubated for 4 days. Then the cells were replated at 5 × 10^5^ cells/150-cm^2^ culture dish (passage 2) and cultured for an additional 14 days. The nucleated cells derived from periosteum, muscle and adipose tissue were isolated and expanded in the same manner as those from synovium.

Bone marrow was aspirated from the tibial tuberosity. Periosteum was peeled off from the tibia. Muscle was obtained from the quadriceps. Adipose tissue was prepared from the subcutaneous fat around the knee. Nucleated cells from the bone marrow were isolated with a density gradient (Ficoll-Paque; Amersham Biosciences, Uppsala, Sweden).

### Colony-formation assay

Nucleated cells derived from synovium were plated at 0.5, 5, 50 and 500 × 10^3^ cells/60-cm^2^ dish, cultured for 14 days, and stained with crystal violet. The optimal initial cell density was determined based on the following criteria: (a) the colony size was not affected by contact inhibition, and (b) the greatest number of colonies was obtained. We then harvested the cells plated at optimal densities from the remaining dishes and expanded them as mentioned above.

### In vitro proliferation assay

Synovial MSCs were plated at 5 × 10^3^ cells/60-cm^2^ dish in complete culture medium and passaged every 14 days. Cells from each passage were harvested and counted with a hemocytometer, and the total accumulated cell number was calculated.

### In vitro differentiation assay

For chondrogenesis, 250 000 cells were placed in a 15-mL polypropylene tube (Becton-Dickinson and Co.) and centrifuged at 450 *g* for 10 min. The pellets were cultured in chondrogenesis medium consisting of high-glucose Dulbecco's modified Eagle's medium (Invitrogen) supplemented with 1 μg/mL bone morphogenetic protein (BMP)-7 (Stryker Biotech, Hopkinton, MA, USA), 10 ng/ mL transforming growth factor (TGF)-β3 (R&D Systems, Minneapolis, MN, USA), 100 nM dexamethasone (Sigma-Aldrich Corp., St Louis, MO, USA), 50 μg/mL ascorbate-2-phosphate, 40 μg/mL proline, 100 μg/mL pyruvate and 1:100 diluted ITS + Premix (6.25 μg/mL insulin, 6.25 μg/mL transferrin, 6.25 ng/mL selenious acid, 1.25 mg/ mL bovine serum albumin and 5.35 mg/mL linoleic acid; BD Biosciences Discovery Labware, Bedford, MA, USA). For microscopy, the pellets were embedded in paraffin, cut into 5-μm sections, and stained with toluidine blue (17–19).

For adipogenesis, cells were cultured in adipogenic medium, which consisted of complete medium supplemented with 100 nM dexamethasone (Sigma-Aldrich Corp.), 0.5 mM isobutyl-methylxanthine (Sigma-Aldrich Corp.) and 50 μM indomethacin (Wako), for 21 days. The adipogenic cultures were fixed in 4% paraformaldehyde and then stained with fresh Oil Red O solution (20).

For calcification, cells were cultured in calcification medium, which consisted of a complete medium of 1 nM dexamethasone, 20 mM (β-glycerol phosphate (Wako) and 50 μg/mL ascorbate-2-phosphate (Sigma-Aldrich Corp.), for 21 days. The cells were fixed in 4% paraformaldehyde and stained with 0.5% Alizarin Red solution (21).

### DiI labeling

Synovial MSCs were resuspended at 1 × 10^6^ cells/ mL in αMEM without FBS, and a fluorescent lipophilic tracer, DiI, was added at a final concentration of 5 μL/mL. After incubation for 20 min at 37°C and two washings with phosphate-buffered saline (PBS), DiI-labeled cells were resuspended in 100 μL culture medium (22).

### Experimental set-up

**Figure 2:**
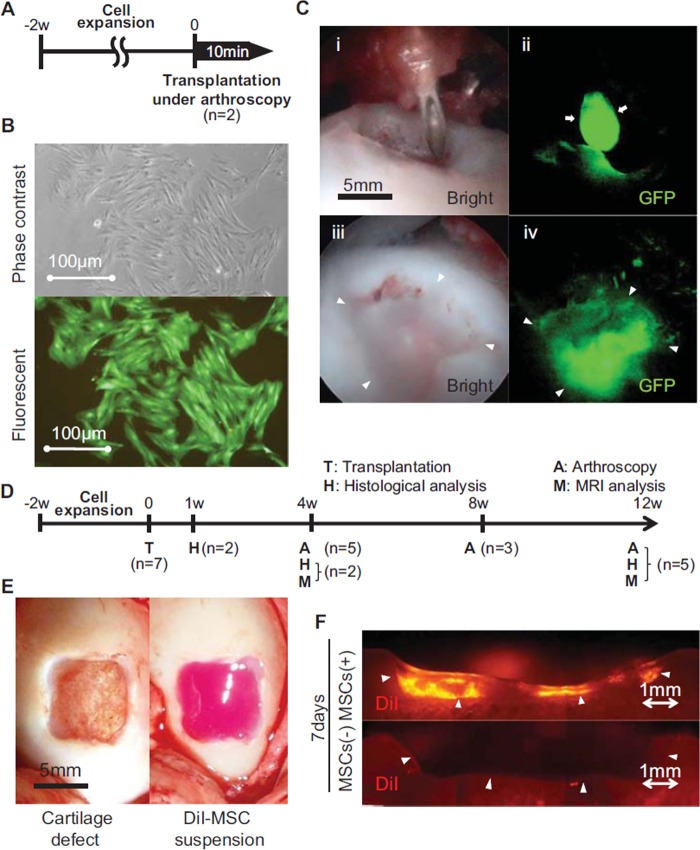
Experimental set-up and local adherent technique for MSCs transplantation. (A) Schematic drawing for arthroscopic transplantation and detection of GFP MSCs. (B) Synovial MSCs from the transgenic GFP pig used to visualize delivery and adhesion of cells in the defect under phase–contrast and fluorescent illumination. (C) Arthroscopic view during transplantation of GFP MSCs into the cartilage defect. Arrows indicate the MSCs suspension leaving the needle. Arrowheads indicate the margin of the cartilage defect. (D) Schematic drawing for histological, MRI and other arthroscopic analyses. (E) Full-thickness cartilage defect (left) and DiI-labeled MSC suspension dropped into the defect (right). (F) Fluorescent images of cartilage defect sections 7 days after transplantation of DiI-labeled MSCs.

The first pig was used for anatomical study and harvesting mesenchymal tissues to stock the MSCs for further analyses. When pigs for the *in vivo* study were prepared, cryopreserved synovial MSCs were thawed and expanded 2 weeks before transplantation. On the day of transplantation surgery, all colony-forming cells were harvested and suspended in 100 μL culture medium and transplanted as described. Four pigs were used for an early adhesion assay with transplantation of GFP porcine synovial MSCs (*n* = 2) (Figure 2A) and DiI-labeled MSCs (*n* = 2). Other pigs were analyzed by arthroscopy every month, and two pigs were sacrificed at 1 month after treatment for histological, macroscopical and MRI analyses. Five pigs were sacrificed at 3 months after treatment and analyzed by histology and MRI (Figure 2D).

### Transplantation of synovial MSCs into the cartilage defects

All pigs underwent general anesthesia, and the medial femoral condyle was approached through a medial parapatellar incision. Full-thickness osteochondral defects (8 × 8 mm square and 2 mm deep; approximately 1.5 mm cartilaginous and 0.5 mm bony part) were created with various sizes of drills in the weight-bearing area of the medial femoral condyles in both knees, 10 mm below the terminal ridge. When the defects were created, bleeding was not observed, and a procedure to stop bleeding from the bottom of the defect was not required.

The right knee of each pig was treated with MSCs and the left knee served as a vehicle internal control. The MSCs were harvested and collected from the culture dishes several hours before transplantation, and harvested MSCs were suspended in a 50-mL conical tube containing 40 mL culture medium. Just before the transplantation, the tube was centrifuged for 5 min at 1500 r.p.m., and the supernatant was removed. Centrifuged MSCs were suspended in 100 μL culture medium. The transplanted cell number was a maximum of 5.3 × 10^7^, a minimum of 2.2 × 10^7^, and on average 3.8 × 10^7^.

The cartilage defect was faced upward, and its position was held manually. A suspension of prepared MSCs in 100 μL culture medium was placed into the defect through an 18-gauge needle. Culture medium alone (100 μL) was placed into the defects in the left knee in the same manner. After 10 min, the incisions were closed without washing the inside of the knee joint. After the anesthetic wore off, the pigs were allowed to walk freely without fixation. To reduce the risk of infection, we avoided the use of an immune suppressor.

For euthanasia, an overdose intravenous injection of KCl was used under adequately deep general anesthesia. For macroscopic analyses, all samples at 1 month (*n* = 3) and 3 months (*n* = 5) were evaluated with the International Cartilage Repair Society (ICRS) macroscopic score (23) (see the supplementary tables).

### Arthroscopy

All knees were observed with arthroscopy (Linvatec 8180A camera console surgical video equipment, with LIS8430 for the light source; Zimmer Inc., Warsaw, IN, USA) at 1, 2 and 3 months after transplantation. An arthroscope, a probe and a shaver system were inserted through longitudinal incisions at the medial and lateral sides of the patella tendon. All arthroscopic observations were evaluated by Oswestry arthroscopy score (23) (see the supplementary tables). For arthroscopic observation of GFP MSCs, a newly developed fluorescence arthroscope (Olympus Medical Systems Corp.,Tokyo, Japan) was used.

### Histological analyses

The samples were cut into a thickness of a 15 mm square with 5 mm containing a defect, fixed in 4% paraformaldehyde, and decalcified with 0.5 M ethylene diamine tetra acetic acid (EDTA; pH 7.5) for 3 days at 4°C. Paraffin sections were stained with Safranin O. All samples were evaluated with a modified Wakitani score (11) (see the supplementary tables).

### dGEMRIC

Before histological analyses, medial femoral condyles were collected and pre-contrast MRI was performed. An MRI system at 1.5 Tesla (Signa HDx; GE Healthcare, Chalfont St Giles, UK) was used with a custom-made micro-imaging coil. Each specimen was pre-treated with 0.5 mM gadopentate dimeglumine (Gd-DTPA^2−^; Magnevist®; Schering, Berlin, Germany) in 0.9% normal saline overnight at 4°C with continuous stirring. The next day the samples were removed from refrigeration, and post-contrast MRI was performed at room temperature. R1 was defined as the reciprocal of the T1 value. The R1 measurement was performed using a fast-spin echo inversion-recovery (FSE-IR) sequence (2400 ms repetition time, 18 ms echo time, six inversion times of 50–2000 ms, 30 × 30 mm field of view, 1.0-mm section thickness, 512 × 512 matrix). The difference between the pre-Gd-enhanced R1 value and the post-Gd enhanced R1 value (ΔR1) indicated the glycosaminoglycan (GAG) concentration (14). Color-coded ΔR1-calculated heat maps of the cartilage were generated using MATLAB (Mathworks, Natick, MA, USA) with a mono-exponential curve fit. Blue represents a high content of GAG, and red a low content. For R1 measurements, the region of interest (ROI) for repaired tissue was defined as the area where both sides were connected between native and repaired cartilage; the bottom was the interface between bone and repaired cartilage, and the top was the superficial surface of the repaired cartilage. The ROI for native cartilage was drawn over the full-thickness weight-bearing areas of the femoral con-dyle at both sides of the repair site, about 3 mm from the lateral edge of the repair site (14,15).

### Statistical analyses

To assess differences, Wilcoxon rank-sum tests were used except for MRI analysis. For MRI analysis, the paired t-test was used. A value of *P<* 0.05 was considered significant.

## Results

### Characteristics of porcine synovial cells as MSCs

**Figure 1:**
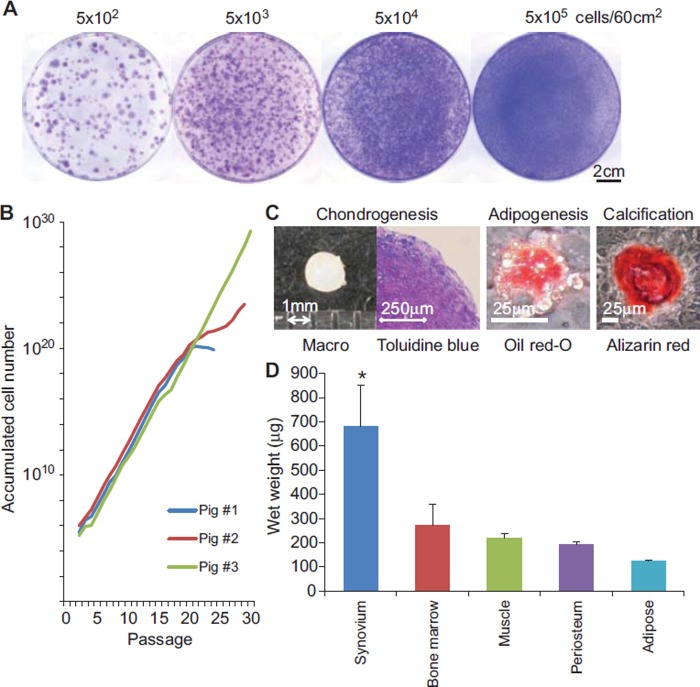
Characteristics of porcine synovial MSCs. (A) Colony formation. (B) Proliferation. (C) *In vitro* chondrogenesis, adipogenesis and calcification. (D) Comparison of the chondrogenic potential among MSCs derived from various mesenchymal tissues. *P< 0.05 (*n* = 5) between synovium and each of the other tissues by Wilcoxon rank-sum test.

The initial cell-plating density to produce the optimal colony number was determined to be 5 × 10^3^ cells/60-cm^2^ dish (Figure 1A). Three cell lineages derived from three different pigs maintained their proliferation potential over 20 passages (Figure 1B). Colony-forming cells derived from porcine synovium displayed a trilineage potential, differentiating into chondrocytes and adipocytes, and osteocytes, when cultured in their respective differentiation media (Figure 1C). *In vitro* chondro-genesis assays demonstrated that cartilage pellets of colony-forming cells derived from synovium were the heaviest among those derived from the other mesenchymal tissues (Figure 1D). These results indicated that colony-forming cells derived from porcine synovium had similar characteristics to those of MSCs, and the highest chondrogenic potential compared with cells derived from the other tissues examined.

### Local adherent technique for transplantation of MSCs

After expanding for 14 days (Figure 2A), colony-forming cells derived from synovium of the transgenic GFP pig expressed GFP (Figure 2B). A drop of *MSC* suspension through a needle (Figure 2Ci) could be detected with the GFP arthroscopy system (Figure 2Cii). After placement of the *MSC* suspension for 10 min, the bottom of the cartilage defect looked foggy (Figure 2Ciii) and GFP MSCs were still detected in the cartilage defect (Figure 2Civ), even though the irrigation fluid was flushed from the tip of the arthroscope (see the supplementary movies). DiI-labeled MSCs were also traced (Figure 2D, E) and remained in the cartilage defect at 7 days (Figure 2F), but they could not be found at 1 and 3 months.

### Arthroscopic and macroscopic observation

**Figure 3:**
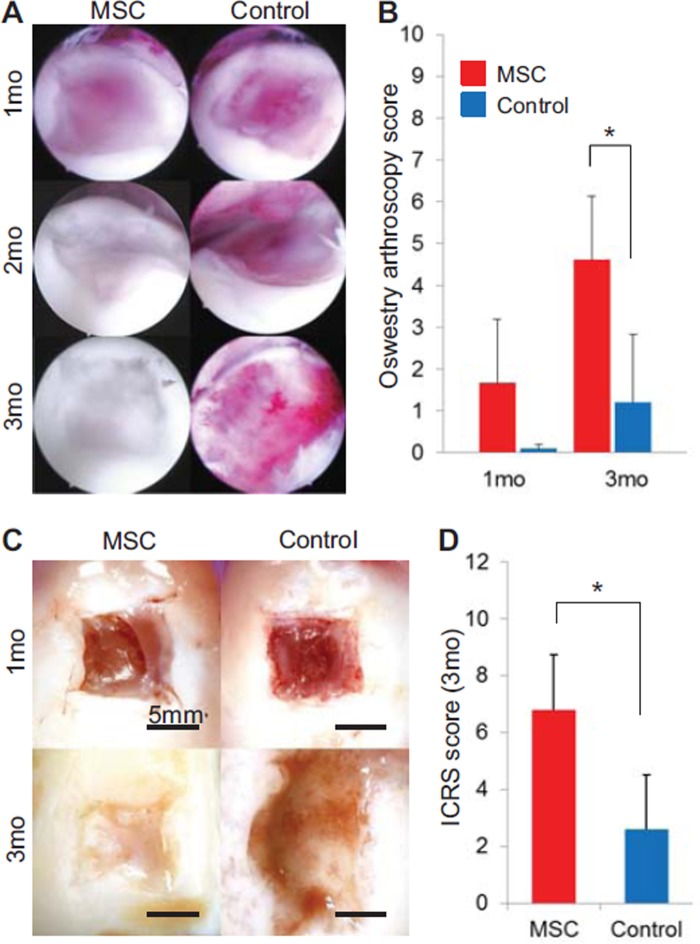
Arthroscopic and macroscopic analyses of cartilage defects with and without transplanted MSC. (A) Sequential arthroscopic view at 1, 2 and 3 months. (B) Quantification of arthroscopic view of cartilage defect. *P< 0.05 by Wilcoxon rank-sum test. (C) Representative macroscopic features. (D) Quantification of macroscopic features of cartilage defect. *P< 0.05 by Wilcoxon rank-sum test.

At 1 month, a thin membrane covered the cartilage defects only in the *MSC*-treated knees (Figure 3A). At 2 months, a thicker white membrane covered the defects in the *MSC*-treated knees, while the cartilage defects were enlarged in the control knees. At 3 months, the defects were covered with cartilage tissue in the *MSC*-treated knees. In contrast, the defects were further enlarged in the control knees. Arthroscopic observation was easier in the *MSC*-treated knees at all time-points because intra-articular adhesion and synovial hypertrophy were less in the *MSC*-treated knees compared with the control knees. The Oswestry arthroscopy score improved over the course of time, and a significant difference between the two groups was observed at 3 months (Figure 3B). Similar results were obtained with the macroscopic evaluation (Figure 3C). The ICRS score for macroscopic observation was significantly higher in the *MSC*-treated knees than in the control knees (Figure 3D). We found no complications throughout this cell transplantation study in the knees examined.

### Histological analyses

**Figure 4:**
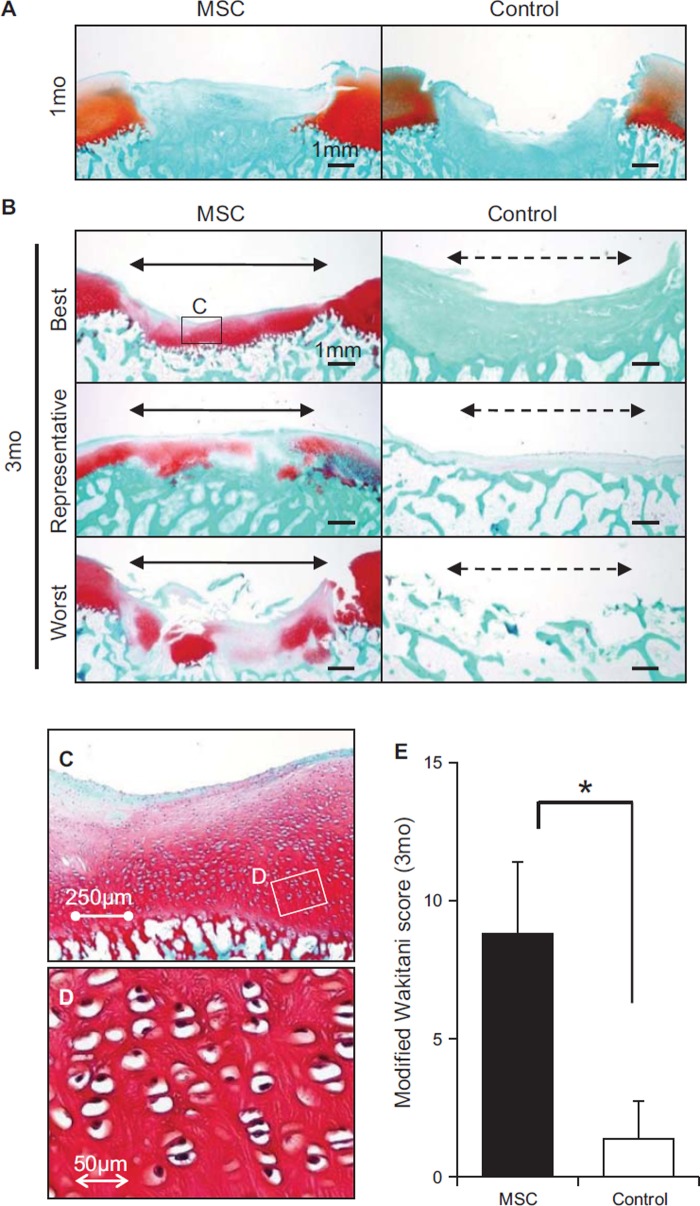
Histological analyses of cartilage defect transplanted with MSCs. (A) Representative sections stained with Safranin O at 1 month. Red indicates extracellular matrix, and blue indicates cancellous bone. (B) Example sections of the best, representative and worst outcomes in the MSC-treated knees at 3 months and in the control from the opposite sides. Borders of the original defect are shown by both arrowheads. (C) Magnified histology of the indicated area. (D) High magnification of the indicated area. (E) Quantification of histologies of cartilage defect. *P< 0.05 by Wilcoxon rank-sum test.

At 1 month, membranous tissue completely covered the defects only in the *MSC*-treated knees (Figure 4A). At 3 months, newly synthesized cartilage matrix was observed in every sample in the *MSC*-treated knees. In contrast, there was no cartilage matrix in the control knees (Figure 4B). Furthermore, cartilage defects were further enlarged in the control knees. Higher magnified observations demonstrated a columnar arrangement of chondrocytes with lacunae in the repaired cartilage in the *MSC*-treated knees (Figure 4C, D). The modified Wakitani score for histological analysis of cartilage repair was significantly higher in the *MSC*-treated knees than in the control knees at 3 months (Figure 4E).

### dGEMRIC

**Figure 5:**
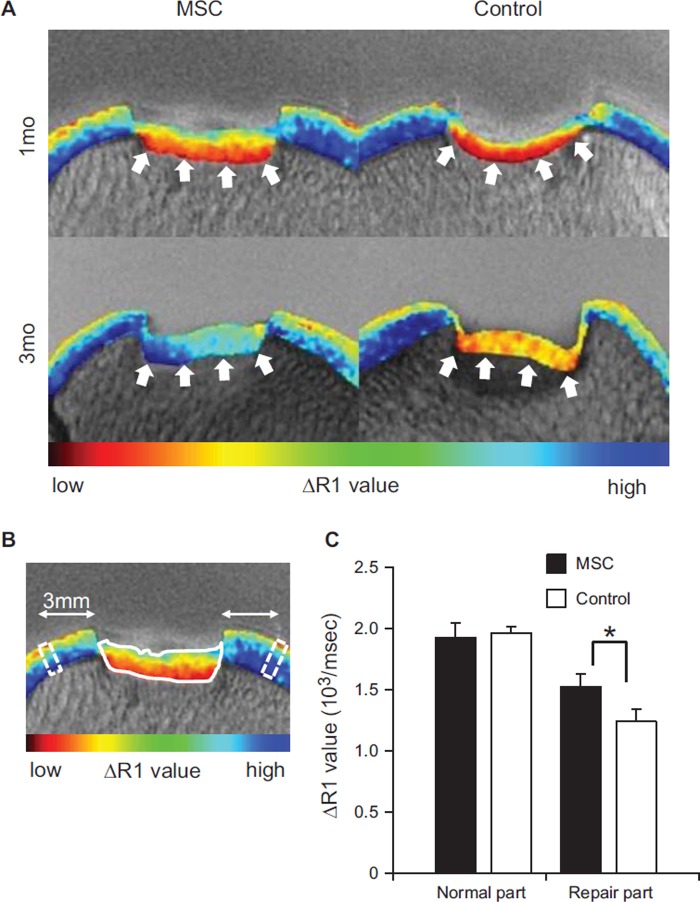
Evaluation with dGEMRIC. (A) Representative images. Arrows indicate the bottoms of the repair tissue. (B) ROI for repaired cartilage (solid-line area) and for native cartilage (dotted-line areas). (C) Quantification of R1 values at 3 months. *P< 0.05 by paired t-test.

The cartilage defects showed predominantly red (lower glycosaminoglycan concentration) in both the *MSC* and control knees at 1 month (Figure 5A). At 3 months, they changed to blue (higher glycosaminoglycan concentration) in the *MSC*-treated knees, while remaining red in the control knees. The average R1 value for ROI (Figure 5B) was higher in the *MSC*-treated knees than in the control knees (Figure 5C).

## Discussion

One of the principal findings of the study was the high chondrogenic potential of MSCs from synovium in pigs. In this study, *in vitro* chondrogenesis assays demonstrated that cartilage pellets of MSCs from synovium were heavier than those from bone marrow, muscle, periosteum and adipose tissue in pig. We have reported similar results previously in humans (4), rats (5) and rabbits (22). These findings suggest that MSCs derived from synovium have a high chondrogenic potential irrespective of animal species.

The *in vitro* chondrogenic potential was evaluated by the weight of the pellet. During *in vitro* chondrogenesis of MSCs, the pellets increased in size and weight. In contrast, the DNA yield per pellet decreased over time. The radioactivity per DNA in the cells, assessed by pre-labeling with 3H-thymidine, was stable during *in vitro* chondrogenesis of MSCs. Consequently, the increase in pellet size could be attributed to the production of extracellular matrix (ECM) and not to the proliferation of the cells (19,24). Pellet weight is always correlated with the expression of cartilage-related mRNA, such as COL2A1, with proteoglycan staining by Safranin O, type II collagen by immunostaining, and protein expression of chondroitin 4-sulfate by enzyme-linked immunosorbent assay (ELISA) (4–7, 17–19, 25). Furthermore, the results of *in vitro* chondrogenesis reflected the results of *in vivo* chondrogenesis in that undifferentiated MSCs were transplanted into cartilage defects, and cartilage matrix production by MSCs was evaluated after 4 weeks in rabbits (6). All the results demonstrate that the weights of the pellets are quantitative indicators for chondrogenesis of MSCs.

*In vitro* chondrogenesis appears to be most successful when a combination of dexamethasone, TGF-β and BMP is used in MSCs derived from bone marrow (18), synovium (19), muscle (26), periosteum (27) and adipose tissue (28). However, our current results do not exclude the possibility that a different combination of growth factors may induce a more effective chondrogenesis dependent on *MSC* sources.

To track the cells, we used both GFP and DiI systems. The use of GFP cells is advantageous in that dead GFP cells are not detected. In this study, GFP synovial MSCs were derived from the Jinhua pig, and the recipients used were Mexican hairless pigs. This was a major mismatch transplantation model, because Jinhua and Mexican hairless pigs have a high independency of gene profile as a result of inbreeding (29). Therefore, the analysis of transplantation of GFP cells was limited for the observation of arthroscopic transplantation of synovial MSCs, because we wanted to avoid the possibility of an immune reaction after adherence of the cells. The use of GFP cells is disadvantageous in that GFP is often undetectable after processing for histology, especially in the case of paraffin embedding (30). To solve these problems, we used the DiI system to track the transplanted cells.

For histological and other analyses, we created cartilage defects and left the suspension of MSCs on the defects for 10 min in an open arthrotomy. For GFP analysis, after the cartilage defects were created in an open arthrotomy, the joint capsule and skin were sutured, then the suspension of MSCs was placed on the defects through the needle while we observed the defect with an arthroscope, and the suspension was left for 10 min. Fluorescence arthroscopy demonstrated that GFP MSCs remained in the cartilage defects, even though the irrigation fluid was flushed from the tip of the arthroscope. This indicates that the method we used makes it possible to transplant MSCs into the cartilage defects through a small incision by arthroscopy, with minimal invasiveness. Although a GFP-detecting endoscopy system for the airway has been reported previously (31), this system still seems to be unpopular. Our study is the first report demonstrating GFP cells in joints with arthroscopy.

In this study, the number of MSCs adhering to the cartilage defect was not quantified. In our previous *ex vivo* study using human and rabbit samples, a suspension of synovial MSCs was placed on the full-thickness defect of the articular cartilage fragment, and approximately 60% of the cells were attached to the defect within 10 min (11). A recent study reported that the addition of magnesium to the cell suspension increased the number of synovial MSCs attached to the cartilage defect *in vitro* and *in vivo* (32). In our pig study, the medium for *MSC* suspension contained 1 mM magnesium, and we estimated that more than 60% of the cells adhered to the cartilage defect.

The cartilage defect we created might be better called an osteochondral defect rather than a cartilage defect. We tried to create a full thickness cartilage defect, but it was not technically easy to do with precision. Therefore, we preferred to create the osteochondral defect in order to be sure all the cartilage was removed, because any remaining cartilage would affect the outcome of this study. We also thought that if we could repair an osteochondral defect with our method, we could also repair a full thickness cartilage defect through further abrading of the full thickness cartilage defect to create an osteochondral defect.

By penetrating the subchondral bone, host bone marrow MSCs would have migrated into the defect. Because bone marrow MSCs also have chondrogenic potential (33), the effect of bone marrow MSCs would not have been negligible in our study. However, we were able to demonstrate the higher effect of synovial MSCs, because the control defects were not repaired at all. The depth of the osteochondral defect may have affected the result of the repair. Chang *et al*. (34) compared the histological score of the spontaneous repair of the defect between a 2-mm and 5-mm depth of osteochondral defect in pigs for 36 weeks, and the score of the 2-mm defect was better than that of the 5-mm osteochondral defect. In our study, a 2-mm osteochondral defect consisting of 1.5 mm in the cartilage and 0.5 mm in the subchondral bone was created, and the influence of the subchondral bone defect would have been less than that when the subchondral bone was penetrated deeper.

In this study, DiI-labeled cells were detected at 1 week, but not at 4 and 12 weeks. The process of cartilage repair was observed within at least 3 months. These findings suggest that transplantation of synovial MSCs secretes some trophic factors to enhance cartilage repair rather than directly differentiating into chondrocytes. According to our recent report, in a co-culture of rat nucleus pulposus cells and human synovial MSCs, a species-specific microarray revealed that gene profiles of the nucleus pulposus were altered markedly, with suppression of genes related to matrix degradative enzymes and inflammatory cytokines (35). Identification of the trophic factors by synovial MSCs in a cartilage defect model is required in a future study.

We have shown that transplantation of synovial MSCs into cartilage defect promotes cartilage repair in pigs. To the best of our knowledge, only Ando *et al*. (36) have previously reported the effect of transplantation of synovial MSCs into cartilage defects in a pig model. They cultured synovial MSCs at a high density in growth medium containing ascorbate 2-phosphate, to form a complex of the cultured cells and the extracellular matrix. After detaching the tissue-engineered construct by application of shear stress using gentle pipetting, the constructs were implanted into the cartilage defect (36). Comparing Ando *et al*.'s study (36) and ours, our method is simpler, and we provide several kinds of novel information during the process of cartilage repair.

We have reported previously that placing a synovial *MSC* suspension on the osteochondral defect for 10 min promotes cartilage regeneration in rabbits. Histological analyses demonstrated that the osteochondral defect was initially filled with cartilage matrix at 4 weeks, then the border between the bone and cartilage moved upward, and finally the thickness of the regenerated cartilage became similar to that of the neighboring cartilage in rabbits (11,32). In the pig study, after transplantation of synovial MSCs, the cartilage defect was first covered with a membrane at 4 weeks, then the cartilage matrix emerged, although the repair of the subchondral bone was not observed. These findings may indicate different processes of cartilage repair between rabbits and pigs.

After placement of the *MSC* suspension, consisting of on average 38 million cells in 100 μL, for 10 min, although the inside of the knee joint was filled with irrigation fluid flushed from the tip of the arthro-scope, the bottom of the cartilage defect looked foggy through conventional light arthroscopy (Figure 2Ciii). This was possible because the cartilage defect was mostly covered with synovial MSCs. The color of the suspension of synovial MSCs was similar to that of the cartilage defect after placement of the *MSC* suspension for 10 min, which supports our speculation. For clinical application, we can guess the existence of MSCs without labeling, by arthroscopic observation if a high concentration of *MSC* suspension is prepared.

dGEMRIC requires more effort than conventional MRI because it requires twice as many imagings both before and after contrast agent administration. However, dGEMRIC can provide information about the thickness of repaired cartilage and glycosaminoglycan concentration (14,15). In this study, we confirmed the usefulness of dGEMRIC for cartilage repair. To the best of our knowledge, this is the first study to analyze porcine cartilage repair by dGEMRIC and to compare its histological results.

Although transplantation of synovial MSCs induced cartilage repair compared with control knees, cartilage repair was not yet complete at 3 months. We can suggest three reasons for this. First, 3 months was too short a time to mature the cartilage defect in this model. Even in our rabbit study, it took 6 months to repair the cartilage defect after transplantation of synovial MSCs (22). In porcine studies by others, it seems that cartilage repair was not complete at 6 months after bone marrow MSCs transplantation (37–39). Because of the limitation of our animal facility, we could not perform observations for more than 3 months in this study. Second, we created the cartilage defect in both knees, and all pigs were free in the cage. Therefore, both knees could not avoid bearing weight. Third, allogeneic synovial MSCs were used in this study to prevent variability of porcine MSCs.

However, this study is valuable because we have demonstrated the ability of synovial-derived MSCs to repair cartilage in the porcine knee relative to vehicle-treated knees. Furthermore, the potential problems in this study, as mentioned above, can be overcome if and when this therapy is applied in humans, because weight bearing can be controlled on the treated knee, and autologous cells can be prepared to expand in autologous human serum (7).

**Figure 6:**
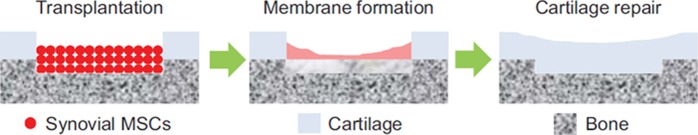
Diagram of the process of cartilage repair. At about 1 month, a membranous layer formed over the defect, and by 3 months cartilage had formed to repair the defect.

In conclusion, an *in vitro* chondrogenesis assay revealed that MSCs from synovium had a higher chondrogenic potential than that from other mesenchymal tissues in pig, as has been found in other species (4,5,22). Through the use of transgenic porcine GFP-expressing synovial MSCs and a new fluorescence arthroscopy system, we were able to visualize the actual delivery and adhesion of the cells in the cartilage defect. We utilized dGEMRIC to obtain detailed serial images of cartilage repair produced by MSCs. Sequential arthroscopic, histological and MRI analyses demonstrated that the cartilage defect was first covered with a membrane, and then the cartilage matrix emerged after transplantation of synovial MSCs (Figure 6).
