# Supplementary material for: Arthroscopic, histological and MRI analyses of cartilage repair after a minimally invasive method of transplantation of allogeneic synovial mesenchymal stromal cells into cartilage defects in pigs
Source: Cytotherapy. 2012 Feb 6;14(3):327–38. doi: 10.3109/14653249.2011.638912 (PMC3296518; doi:10.3109/14653249.2011.638912)
Supplement: Supplemental Tables [file SD1.pdf]

Oswestry Arthroscopy Score

OAS	Points
Graft level with surrounding cartilage	
Level	2
Raised	1
Below	0
Integration with surrounding cartilage	
Complete	2
Minor disruption (<25% of area)	1
Major disruption (>25% of area)	0
Appearance of surface	
Smooth	2
Fine fronds	1
Severe fronds/fibrillation	0
Color of graft	
Pearly, hyaline-like	2
White	1
Yellow bone	0
Stiffness on probing	
Normal compared to adjacent cartilage	2
Softer	1
Very soft/hard	0
Total	0–10

ICRS Macroscopic Score

Cartilage repair assessment ICRS	Points
Degree of defect repair	
In level with surrounding cartilage	4
75% repair of defect depth	3
50% repair of defect depth	2
25% repair of defect depth	1
0% repair of defect depth	0
Integration to border zone	
Complete integration with surrounding cartilage	4
Demarcating border < 1 mm	3
3/4th of graft integrated, 1/4th with a notable border > 1 mm width	2
1/2 of graft integrated with surrounding cartilage, 1/2 with a notable border > 1 mm	1
From no contact to 1/4th of graft integrated with surrounding cartilage	0
Macroscopic appearance	
Intact smooth surface	4
Fibrillated surface	3
Small, scattered fissures or cracs	2
Several, small or few but large fissures	1
Total degeneration of grafted area	0
Overall repair assessment	
Grade I: normal	12
Grade II: nearly normal	11–8
Grade III: abnormal	7–4
Grade IV: severely abnormal	3–1

Modified Wakitani Score

Category	Points
Cell morphology	
Hyaline cartilage	4
Mostly hyaline cartilage	3
Mostly fibrocartilage	2
Mostly non-cartilage	1
Noncartilage only	0
Matrix-staining (metachromasia)	
Normal (compared with host adjacent cartilage)	3
Slightly reduces	2
Markedly reduced	1
No metachromatic stain	0
Surface regularity[a]	
Smooth (>3/4)	3
Moderate (1/2 to 3/4)	2
Irregular (1/4 to 1/2)	1
Severely irregular (<1/4)	0
Thickness of cartilage[b]	
2/3 to 4/3	3
5/3 to 4/3	2
1/3 to 2/3 or >5/3	1
<1/3	0
Integration of donor with host adjacent cartilage	
Both edges integrated	2
One edge integrated	1
Neither edge integrated	0
Total maximum	15

[a]Total smooth area of the reparative cartilage compared with the entire area of the cartilage defect. [b]Average thickness of the reparative cartilage compared with that of the surrounding cartilage.
